# Supplementary material for: Analysis of the Consumption of Sports Supplements in Open Water Swimmers According to the Competitive Level
Source: Nutrients. 2022 Dec 7;14(24):5211. doi: 10.3390/nu14245211 (PMC9783229; doi:10.3390/nu14245211)
Supplement: Supplementary file 1 [file nutrients-14-05211-s001.zip › nutrients-2022730-supplementary.pdf]

ESTUDIO DEL CONSUMO DE SUPLEMENTOS NUTRICIONALES EN NADADORES DE AGUAS ABIERTAS

Estimad@ amig@, estamos realizando un estudio sobre el uso de suplementos en su deporte. Su colaboración nos será de gran ayuda, estándole agradecidos enormemente si de modo altruista decide contestar a estas preguntas. Su identidad permanecerá en el anonimato, y sólo se usarán los datos de manera estadística para conocer la realidad del consumo de suplementos nutricionales en nadadores de aguas abiertas. La Ley Orgánica de Protección de Datos será respetada en todo momento.

El estudio cumple con la Declaración de Helsinki para investigación en humanos y ha sido aprobado por el Comité de Ética de la Universidad de Alicante (Expediente UA 2021-02-01)

No rellenar más de una inscripción por persona y escribir en MAYÚSCULAS todas las respuestas que requieran texto.

*Obligatorio

DATOS INICIALES

1. SEXO *

Marca solo un óvalo.

- ◯ Hombre
- ◯ Mujer

2. EDAD *

____

3. ALTURA APROXIMADA EN CENTÍMETROS (cm) *

En centímetros

____

4. PESO APROXIMADO EN KILOGRAMOS (kg) *

En kilogramos

5. % APROXIMADO DE GRASA (Solo si se sabe la respuesta)

Porcentaje

6. COMUNIDAD AUTÓNOMA DE RESIDENCIA *

Marca solo un óvalo.

- Andalucía
- Aragón
- Principado de Asturias
- Islas Baleares
- Canarias
- Cantabria
- Castilla y León
- Castilla-La Mancha
- Cataluña
- Comunidad Valenciana
- Extremadura
- Galicia
- Comunidad de Madrid
- Región de Murcia
- Comunidad Foral de Navarra
- País Vasco
- La Rioja
- Ciudad Autónoma de Ceuta
- Ciudad Autónoma de Melilla

PRÁCTICA DEL DEPORTE

7. INDICA EL NÚMERO DE AÑOS APROXIMADO QUE LLEVAS FEDERADO *

Marca solo un óvalo.

- 1 año
- 2 años
- 3 años
- 4 años
- 5 años
- 6 años
- 7 años
- 8 años
- 9 años
- 10 o más años

8. INDICA EN EL NIVEL QUE COMPITES ACTUALMENTE *

Señala el de mayor categoría

Marca solo un óvalo.

- Regional
- Nacional
- Continental
- Mundial
- Olímpico
- Otro: ____________________

9. INDICA LOS AÑOS QUE LLEVAS COMPITIENDO EN EL NIVEL SEÑALADO ANTERIORMENTE *

Marca solo un óvalo.

- 1 año
- 2 años
- 3 años
- 4 años
- 5 años
- 6 años
- 7 años
- 8 años
- 9 años
- 10 o más años

ENTRENAMIENTO Y COMPETICIÓN

10. ¿CUÁNTOS DÍAS DE MEDIA DEDICAS AL ENTRENAMIENTO A LA SEMANA? *

APROXIMADAMENTE

Marca solo un óvalo.

- 1 día
- 2 días
- 3 días
- 4 días
- 5 días
- 6 días
- 7 días

11. ¿CUÁNTO TIEMPO, APROXIMADAMENTE, DEDICAS CADA DÍA AL ENTRENAMIENTO? *

Elige el intervalo de tiempo que más se ajuste a tu tiempo de entrenamiento

Marca solo un óvalo.

- <30 minutos
- Entre 30 minutos y 1 hora
- Entre 1 hora y 1,5 horas
- Entre 1,5 horas y 2 horas
- >2 horas

12. SUELES ENTRENAR... *

Elige el intervalo de tiempo que más se ajuste a tu tiempo de entrenamiento

Marca solo un óvalo.

- Por la mañana
- Por la tarde
- Por la mañana y la tarde
- Otro: ____________________

13. ¿CUÁNTAS COMPETICIONES TIENES AL AÑO? *

Elige la opción que más se acerque a la realidad

Marca solo un óvalo.

- 1
- 2
- 3
- 4
- 5
- 6
- 7
- 8
- 9
- 10
- 11
- 12
- más de 12
- Sin frecuencia exacta
- No sabe no contesta

14. ¿EN QUÉ DISTANCIA DE AGUAS ABIERTAS SUELES COMPETIR? (Si son varias, indicar la más frecuente. En caso de elegir Otra, especifica distancias)

Selecciona todos los que correspondan.

- [ ] 5 km
- [ ] 10 km
- [ ] 25 km
- Otro: [ ] ____________________

15. SUELES COMPETIR... *

Elige el intervalo de tiempo que más se ajuste a tu tiempo de entrenamiento

Marca solo un óvalo.

- Por la mañana
- Por la tarde
- Por la mañana y la tarde
- Otro: ______

DIETA

16. ¿SIGUES EN LA ACTUALIDAD ALGÚN TIPO DE DIETA O FORMA CONCRETA DE ALIMENTARSE? *

Marca solo un óvalo.

- Si *Salta a la pregunta 17*
- No *Salta a la pregunta 20*

DIETA CONTINUACIÓN

17. ¿QUÉ TIPO DE DIETA ES? *

Marca solo un óvalo.

- Vegetariana
- Vegana
- Mediterránea
- Ovolactovegetariana
- Paleo
- Disociada
- Macrobiotica
- Flexible
- Cetogénica
- Otro: ______________________

18. ¿POR QUÉ LA HACES? *

Marca solo un óvalo.

- Para cuidar tu salud
- Por estética
- Por enfermedad
- Por rendimiento
- Por religión o creencias
- Otro: ______________________

19. ¿TE ASESORA ALGUIEN O TE APOYAS EN ALGO PARA LLEVAR LA DIETA? *

EN CASO AFIRMATIVO INDICA LA RESPUESTA ADECUADA.

Marca solo un óvalo.

- No
- Dietista-Nutricionista
- Médico
- Farmaceutico
- Entrenado Personal
- Fisioterapeuta
- Alguien sin ningula titulación
- Me apoyo en un libro
- Me apoyo en un blog
- Me apoyo en un amigo
- Me apoyo en Redes Sociales
- Otro: ______________________

SUPLEMENTOS

20. ¿ESTÁS A FAVOR DEL CONSUMO DE SUPLEMENTOS EN LA ACTIVIDAD FÍSICA DENTRO DE LA LEGALIDAD? *

Marca solo un óvalo.

- Si
- No
- No sabes/No contestas

21. ¿HAS CONSUMIDO EN ALGUNA OCASIÓN ALGÚN SUPLEMENTO NUTRICIONAL? *

Marca solo un óvalo.

- Si *Salta a la pregunta 22*
- No

SUPLEMENTOS CONTINUACIÓN

22. SEÑALA CUALES DE ESTOS SUPLEMENTOS CONSUMES HABITUALMENTE DURANTE LA TEMPORADA *

ESTÁN ORDENADOS ALFABÉTICAMENTE. PUEDES SEÑALAR TANTOS CUANTOS CONSUMAS DE MANERA HABITUAL

Selecciona todos los que correspondan.

- [ ] 5-HTP (5-Hidroxitriptofano)
- [ ] ATP
- [ ] Ácido Aspártico
- [ ] Ácido Alfa Lipoico (ALA)
- [ ] Ácido Fosfatídico
- [ ] Ácido Hialurónico
- [ ] Ácidos Grasos ω-3
- [ ] Ácidos Grasos ω-6
- [ ] Ácidos Grasos ω-9
- [ ] Ácido Linoleico Conjugado (CLA)
- [ ] Aceite de Coco
- [ ] Aceite de Hígado de Bacalao
- [ ] Aceite de Lino
- [ ] Aceite de Onagra
- [ ] Aceite de Prímula
- [ ] Amilopectina
- [ ] Aminoácidos esenciales (EEAA)
- [ ] Aminoácidos ramificados (BCAA)
- [ ] Arginina
- [ ] Barritas Energéticas
- [ ] Bebida Isotónica
- [ ] Beta Alanina
- [ ] Bicarbonatos
- [ ] Bloqueadores de Carbohidratos
- [ ] Cafeína
- [ ] Carbohidratos ("Gainers")
- [ ] Carnitina (Acetil-L-Carnitina)
- [ ] Carnitina (L-Carnitina)
- [ ] Cartílago de Tiburón
- [ ] Caseína Micelar
- [ ] Chitosan
- [ ] Ciclodextrinas
- [ ] Cinc

- [ ] Citrulina (Malato o L-citrulina)
- [ ] Complejo Mineral
- [ ] Complejo Vitamínico
- [ ] Condroitina
- [ ] Creatina (Monohidrato)
- [ ] Creatina (Kre-Alkalyn)
- [ ] Creatina (Etil Éster)
- [ ] Curcumina
- [ ] Dextrosa
- [ ] Dimetilglicina
- [ ] Diuréticos
- [ ] Electrolitos (en polvo o pastillas)
- [ ] Epicatequina
- [ ] Epigalocatequina-3-galato (EGCG)
- [ ] Espirulina
- [ ] Fórmulas pre-entreno
- [ ] Fosfato de sodio
- [ ] Ginseng
- [ ] Glicerol
- [ ] Glucosamina
- [ ] Glutamina
- [ ] Greens
- [ ] Guarana
- [ ] Hidrolizado de caseína
- [ ] Hidroximetilbutirato (HMB)
- [ ] Hierro
- [ ] Isomaltulosa
- [ ] Jalea Real
- [ ] Lecitina de Soja
- [ ] Leucina
- [ ] Levadura de Cerveza
- [ ] Magnesio
- [ ] Maltodextrina
- [ ] Melatonina
- [ ] Metil-sulfonil-metano (MSM)
- [ ] Nitrato (zumo de remolacha)
- [ ] Nootrópicos
- [ ] Picolinato de Cromo
- [ ] Polen

- [ ] Potenciador de Testosterona
- [ ] Precursor Hormonal
- [ ] Probióticos
- [ ] Proteína de Carne
- [ ] Proteína de Suero (Whey Protein)
- [ ] Proteína Vegetal (soja, cáñamo…)
- [ ] Quercetina
- [ ] Ribosa
- [ ] Sinefrina (p-sinefrina)
- [ ] Suplemento de cereza ácida
- [ ] Taurina
- [ ] Teanina
- [ ] Teacrina
- [ ] Té verde (completo o extractos)
- [ ] Tribulus
- [ ] Tirosina
- [ ] Triglicéridos de cadena media (MCT)
- [ ] Vitamina C
- [ ] Vitamina D
- [ ] Vitamina E
- [ ] Vitamina K
- [ ] Yohimbina
- [ ] ZMA

Otro: [ ] ____________________

Sin título

23. INDICA QUÉ DÍAS DE PRACTICA DEPORTIVA SUELES CONSUMIRLOS *

Marca solo un óvalo.

- Entrenamiento *Salta a la pregunta 22*
- Competición
- Entrenamiento y competición
- Periodo vacacional o de descanso
- En todos lo casos anteriormente mencionados
- Otro: ______________________

24. INDICA CUANDO SUELES CONSUMIRLOS *

Marca solo un óvalo.

- Antes de la práctica deportiva
- Durante la práctica deportiva
- Después de la práctica deportiva
- En todos los casos anteriores
- Indiferentemente
- Otro: ______________________

25. ¿CON QUÉ FIN LOS CONSUMES? *

Selecciona todos los que correspondan.

- [ ] Para cuidar tu salud
- [ ] Por problemas de salud
- [ ] Por necesidad
- [ ] Por obligación
- [ ] Para mejorar su aspecto físico
- [ ] Para buscar rendimiento deportivo
- [ ] Por paliar algún déficit de la dieta

Otro: [ ] ______________________

26. ¿DONDE SUELES COMPRAR LOS SUPLEMENTOS? *

Selecciona todos los que correspondan.

- [ ] En una Farmacia
- [ ] En un Gimnasio
- [ ] A un Amigo
- [ ] A un Monitor
- [ ] A un Dietista-Nutricionista
- [ ] En un Herbolario
- [ ] En un Centro Comercial
- [ ] En una Parafarmacia
- [ ] En Internet
- [ ] En una Tienda especializada

Otro: [ ] ______________________

27. ¿QUÉ/QUIEN TE MOTIVÓ A TOMARLOS? *

Selecciona todos los que correspondan.

- [ ] Compañero de equipo
- [ ] Entrenador
- [ ] Internet
- [ ] Monitor
- [ ] Publicidad
- [ ] Dietista-Nutricionista
- [ ] Revista especializada
- [ ] Televisión
- [ ] Preparador Físico
- [ ] Médico
- [ ] Amigo

Otro: [ ] ______________________

28. EN GENERAL, ¿CREES QUE TE DIERON RESULTADOS? *

Marca solo un óvalo.

	1	2	3	4	5	
NINGÚN RESULTADO	⬭	⬭	⬭	⬭	⬭	MUCHO RESULTADO

29. SI FUERA EL CASO, ESCRIBE ALGÚN/OS DE LOS SUPLEMENTOS QUE CREES QUE NO TE DIERON NINGÚN RESULTADO

____

30. SI FUERA EL CASO, ESCRIBE ALGÚN/OS DE LOS SUPLEMENTOS QUE CREES QUE SI TE DIERON RESULTADO

31. ¿HAS CONSUMIDO O CONSUMIRÍAS ALGÚN SUPLEMENTO QUE FUERA PERJUDICIAL PARA LA SALUD PERO QUE TE AYUDARA A CONSEGUIR TUS OBJETIVOS? *

Marca solo un óvalo.

- Si
- No
- No sabe/No contesta

32. EN EL CASO QUE LA PREGUNTA ANTERIOR SEA AFIRMATIVA, SEÑALA CUÁL/ES DE LA SIGUIENTE LISTA HAS CONSUMIDO O CONSUMIRÍAS

Selecciona todos los que correspondan.

- [ ] Esteroides anabolizantes
- [ ] Hormona de crecimiento
- [ ] Insulina
- [ ] Efedrina
- [ ] Pseudoanfetaminas

Otro: [ ] ____________________

33. INDICA, DESDE TU PUNTO DE VISTA, EL USO GENÉRICO DE ESTE TIPO DE SUSTANCIAS EN AGUAS ABIERTAS *

Marca solo un óvalo.

	1	2	3	4	5	
NINGÚN USO	( )	( )	( )	( )	( )	MUCHO USO

Este contenido no ha sido creado ni aprobado por Google.

Google Formularios
